# Supplementary figures and images for: Combining inferred regulatory and reconstructed metabolic networks enhances phenotype prediction in yeast
Source: PLoS Comput Biol. 2017 May 17;13(5):e1005489. doi: 10.1371/journal.pcbi.1005489 (PMC5453602; doi:10.1371/journal.pcbi.1005489)

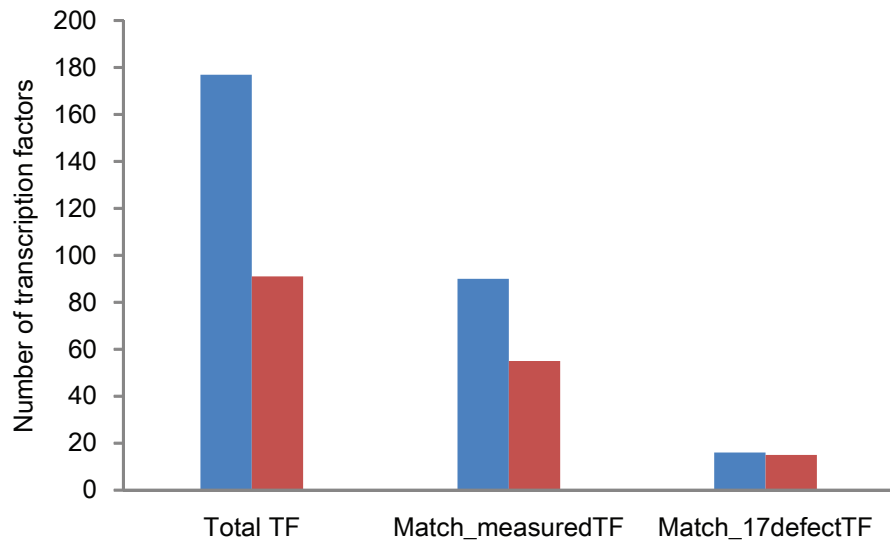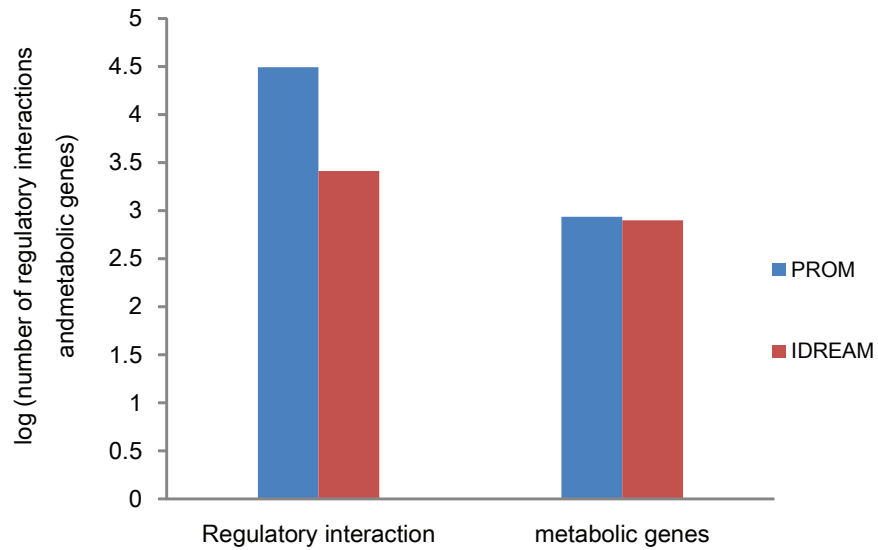

Supplement: S1 Fig — A. The number of transcription factors in PROM and IDREAM. ‘Match_measuredTF’ is the number of TFs having a corresponding phenotype in Fendt’s experiment for 119 TF mutants. ‘Match_17defectTF’ is the number of TFs out of the 17 defect-inducing TFs that are involved in the two integrated models. B. The log value of number of regulatory interactions and metabolic genes in PROM and IDREAM. (PDF) [file pcbi.1005489.s001.pdf]

**A. Predictions by IDREAM  
(Threshold=0.5)**

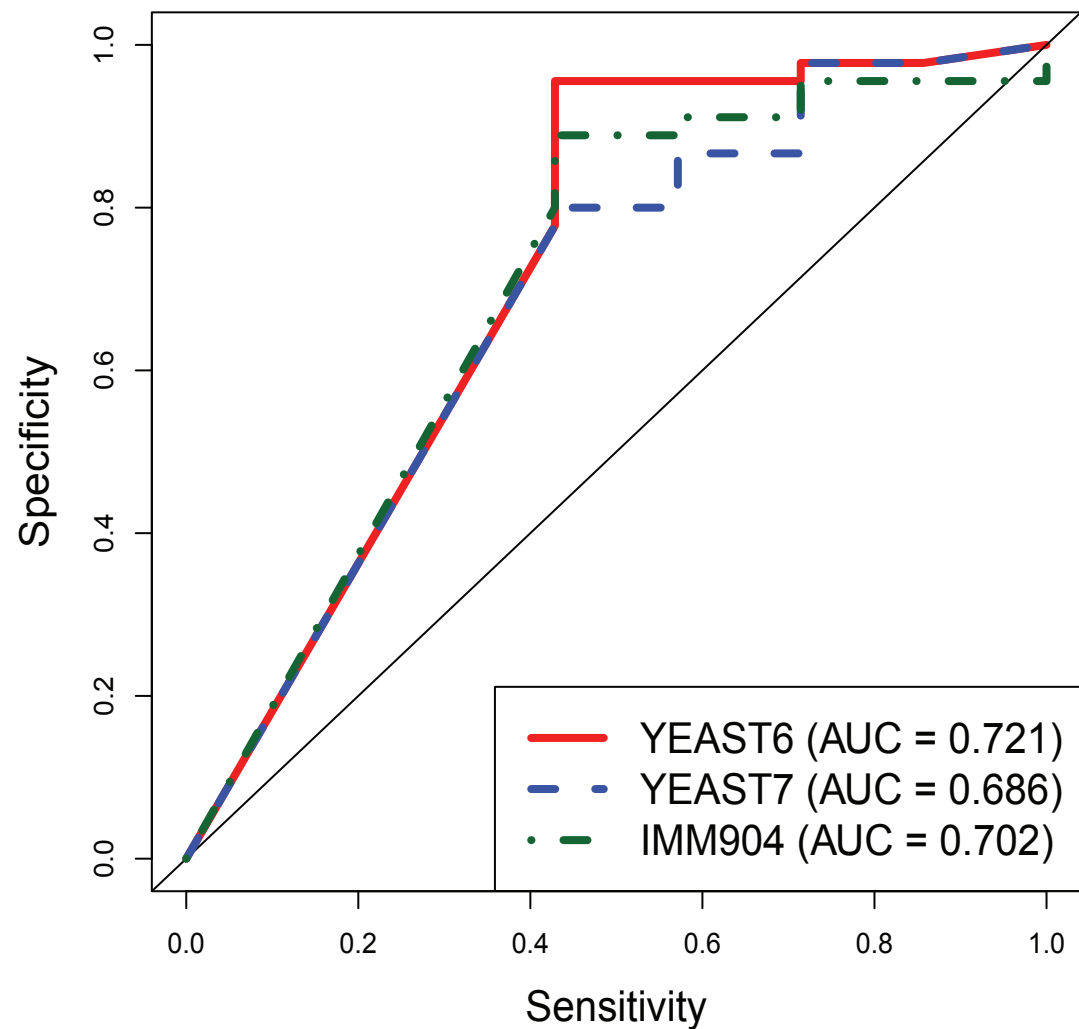

**B. Predictions by PROM  
(Threshold=0.5)**

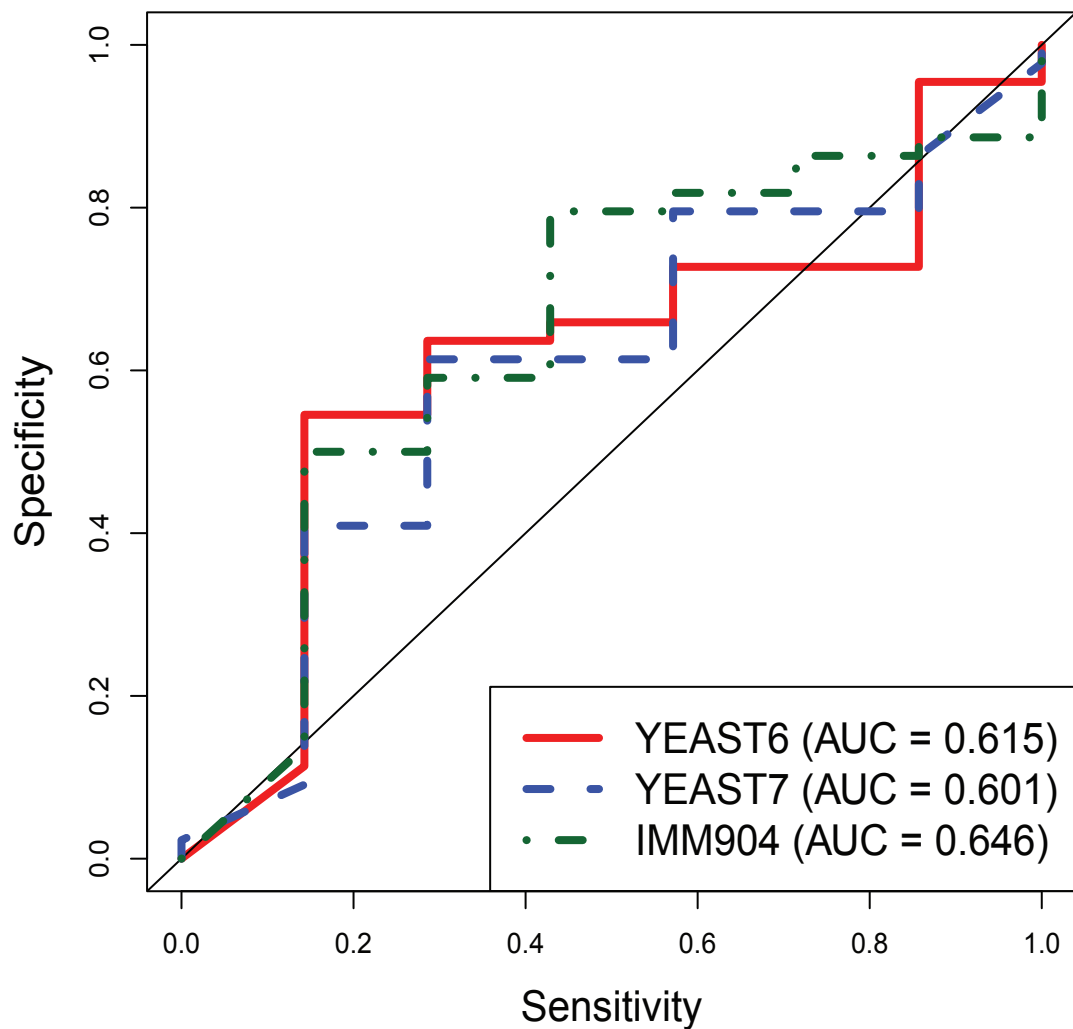

Supplement: S3 Fig — There are no significant differences by the three yeast models. (PDF) [file pcbi.1005489.s003.pdf]

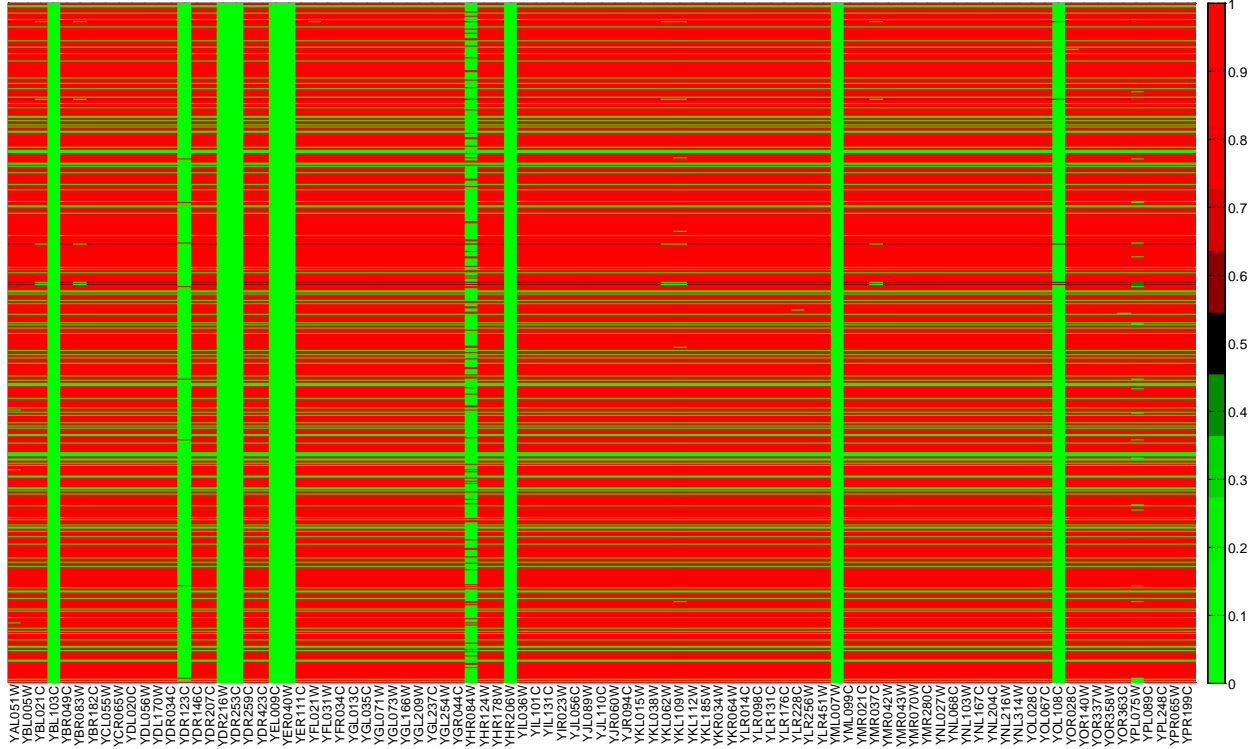

Supplement: S4 Fig — Each row represents a metabolic gene, and each column represents a gene encoding a TF. (PDF) [file pcbi.1005489.s004.pdf]

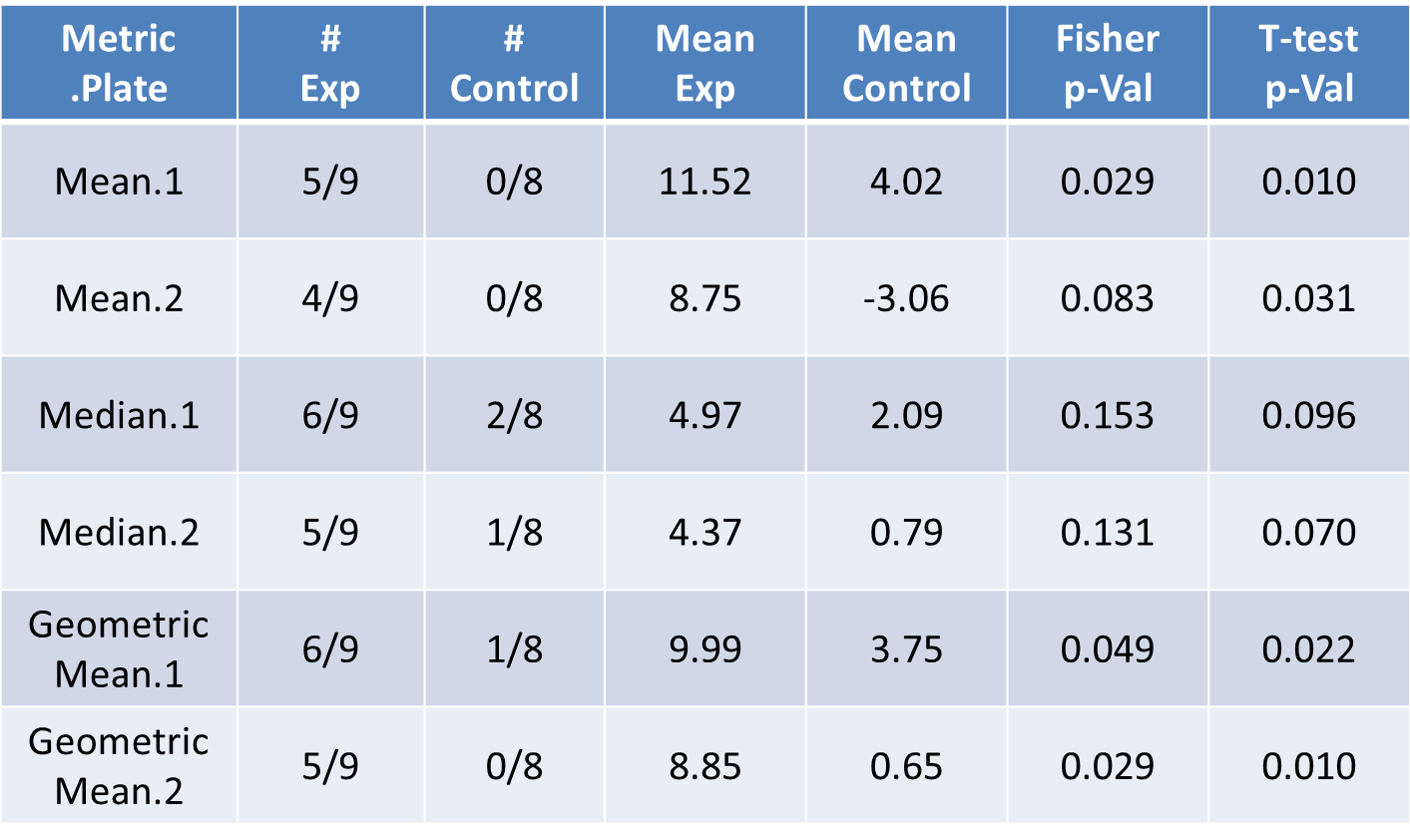

Supplement: S5 Fig — Estimated doubling times inform a confidence score identifying double deletion strains with synthetic growth defects. The first columns shows the metric and plate, such that ‘Mean.1’ means that for replicate 1, the average growth rate of many colonies was estimated using the mean and ‘Geometric Mean.2’ means that for replicate 2, the average growth rate was calculated using the geometric mean. ‘# Exp’ and ‘# Control’ refers to the number of strains in the experiment and control sets (respectively) where the growth rate in the double deletion was significantly less than that expected by adding together the growth rates for single deletions. ‘Mean Exp’ and ‘Mean Control’ are the mean growth decrease in growth rate beyond that expected from adding together the single deletion decreases in growth rate for the experimental and control sets (respectively). ‘Fisher p-Val’ uses a Fisher’s exact test to compare the ‘# Exp’ to the ‘# Control’ while ‘T-test p-Val’ uses the measured magnitudes of the synergistic growth defects. (PNG) [file pcbi.1005489.s005.png]
