# Supplementary material for: Combining inferred regulatory and reconstructed metabolic networks enhances phenotype prediction in yeast
Source: PLoS Comput Biol. 2017 May 17;13(5):e1005489. doi: 10.1371/journal.pcbi.1005489 (PMC5453602; doi:10.1371/journal.pcbi.1005489)
Supplement: S2 Fig — Across 16 different thresholds, the AUC value is significantly higher for IDREAM (mean = 0.67) than PROM (mean = 0.58). (PDF) [file pcbi.1005489.s002.pdf]

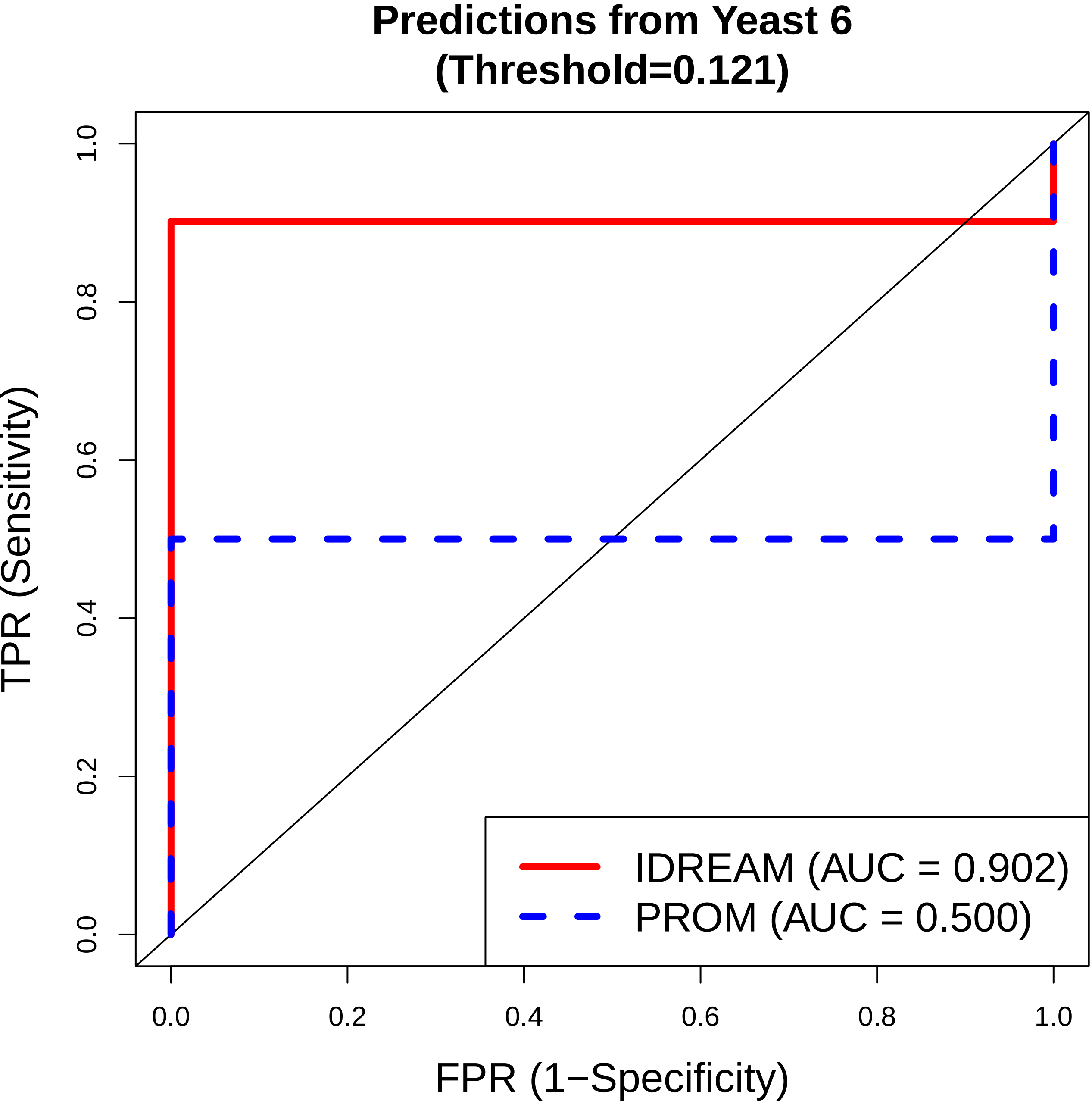

Predictions from Yeast 6
(Threshold=0.121)
TPR (Sensitivity)
0.0
0.2
0.4
0.6
0.8
1.0
FPR (1−Specificity)
0.0
0.2
0.4
0.6
0.8
1.0
IDREAM (AUC = 0.902)
PROM (AUC = 0.500)

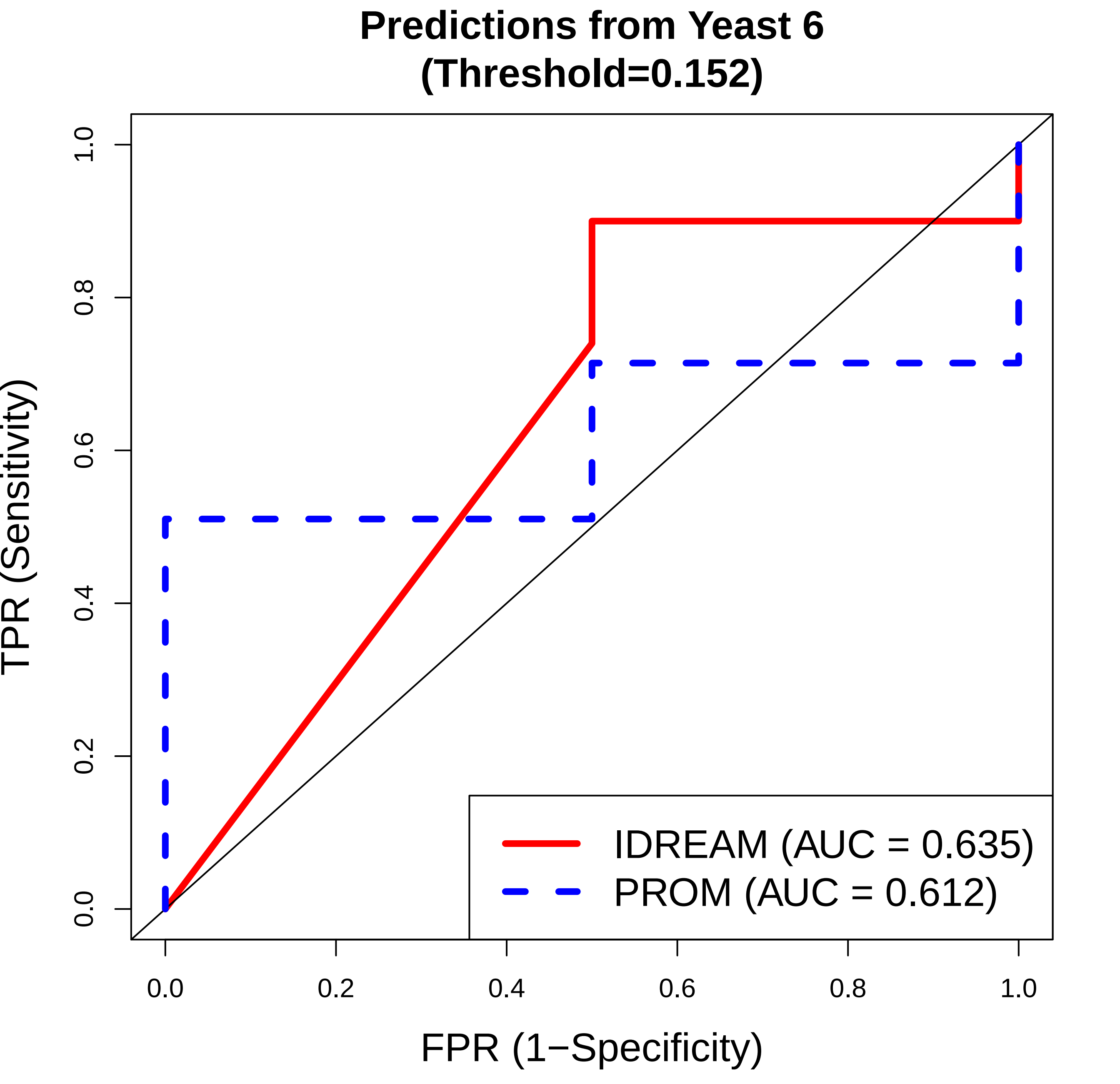
Predictions from Yeast 6
(Threshold=0.152)
TPR (Sensitivity)
FPR (1−Specificity)
0.0
0.2
0.4
0.6
0.8
1.0
IDREAM (AUC = 0.635)
PROM (AUC = 0.612)

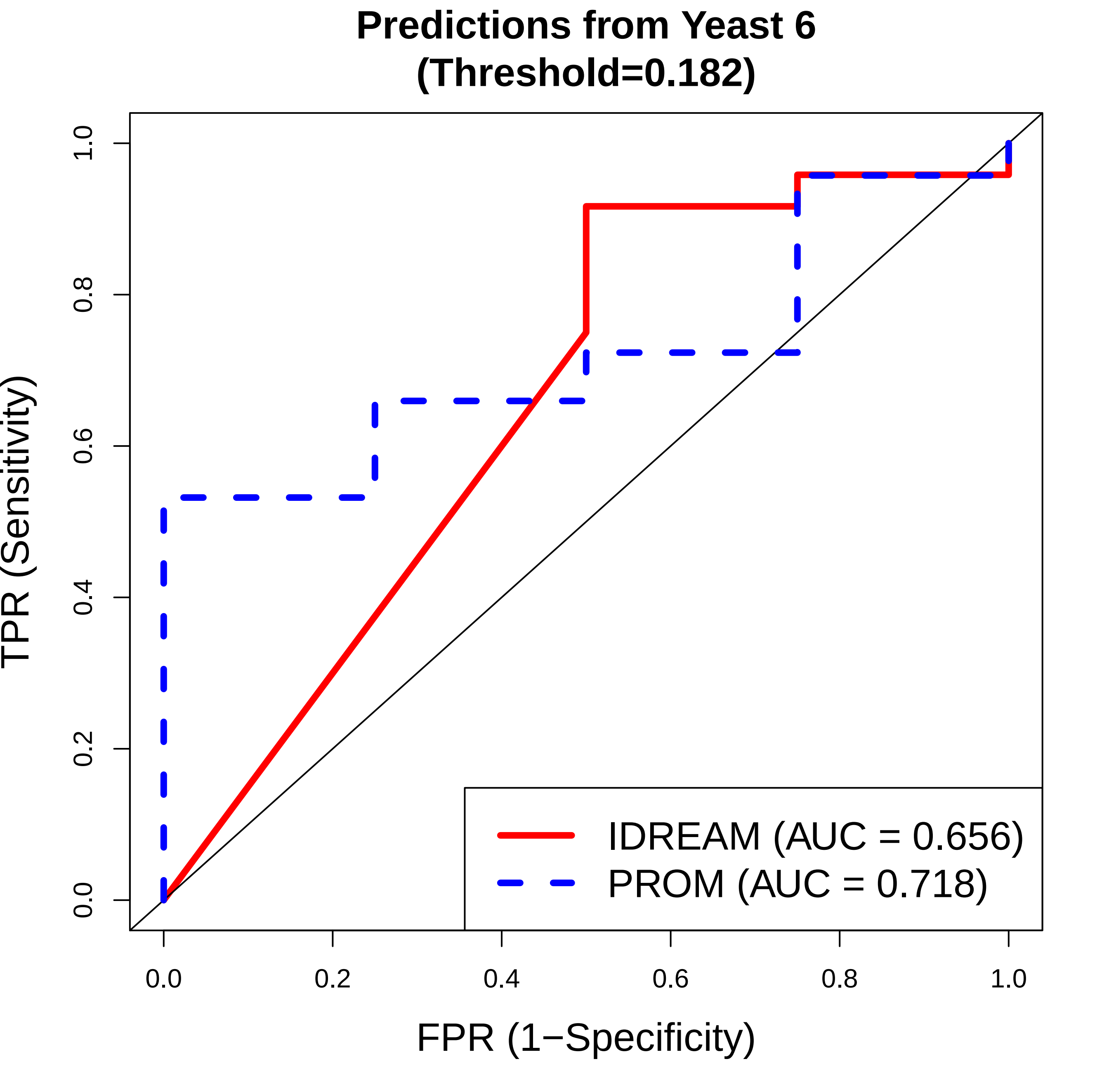

Predictions from Yeast 6
(Threshold=0.182)
TPR (Sensitivity)
FPR (1−Specificity)
0.0
0.2
0.4
0.6
0.8
1.0
IDREAM (AUC = 0.656)
PROM (AUC = 0.718)

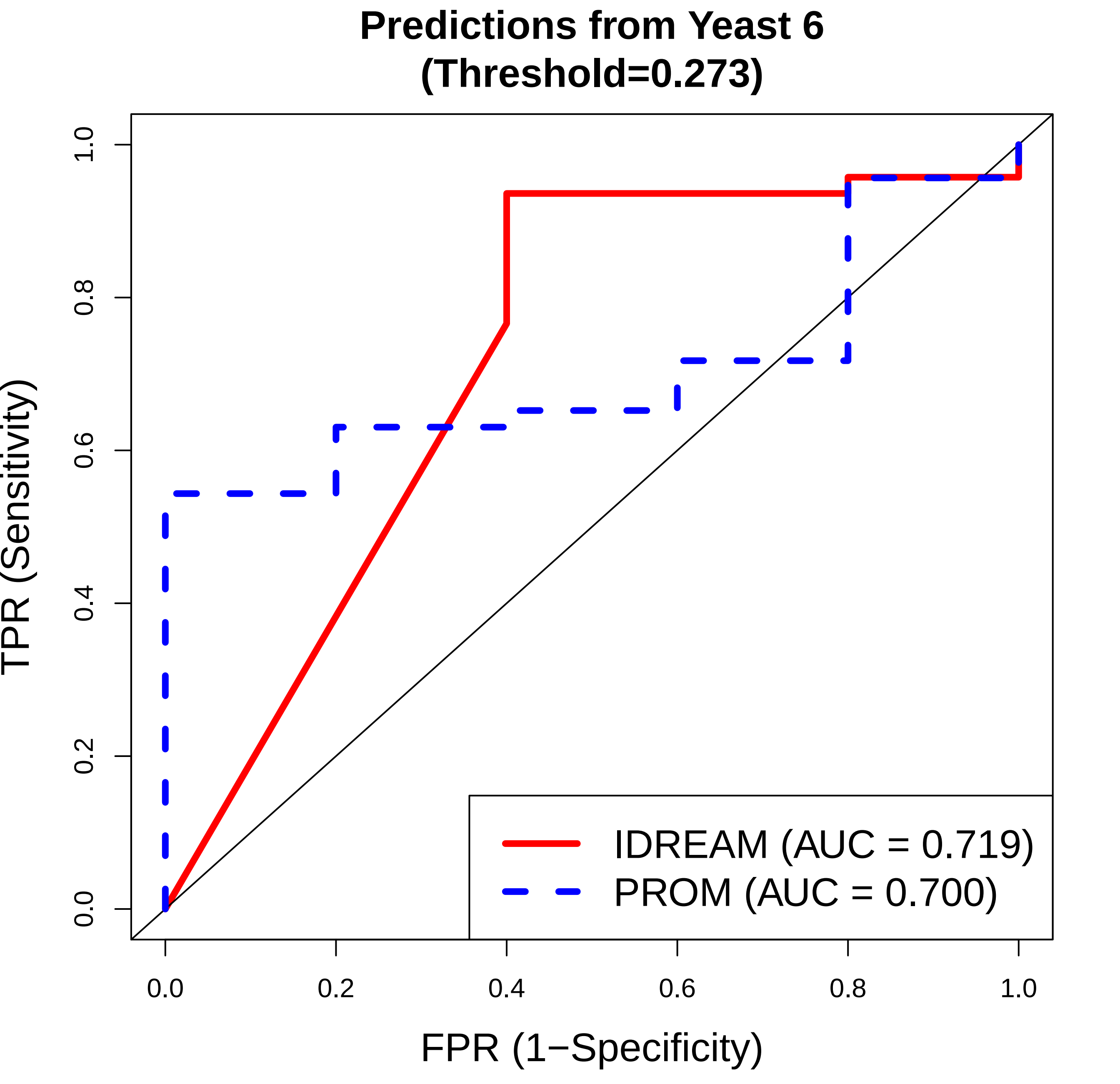
Predictions from Yeast 6
(Threshold=0.273)
TPR (Sensitivity)
FPR (1−Specificity)
0.0
0.2
0.4
0.6
0.8
1.0
IDREAM (AUC = 0.719)
PROM (AUC = 0.700)

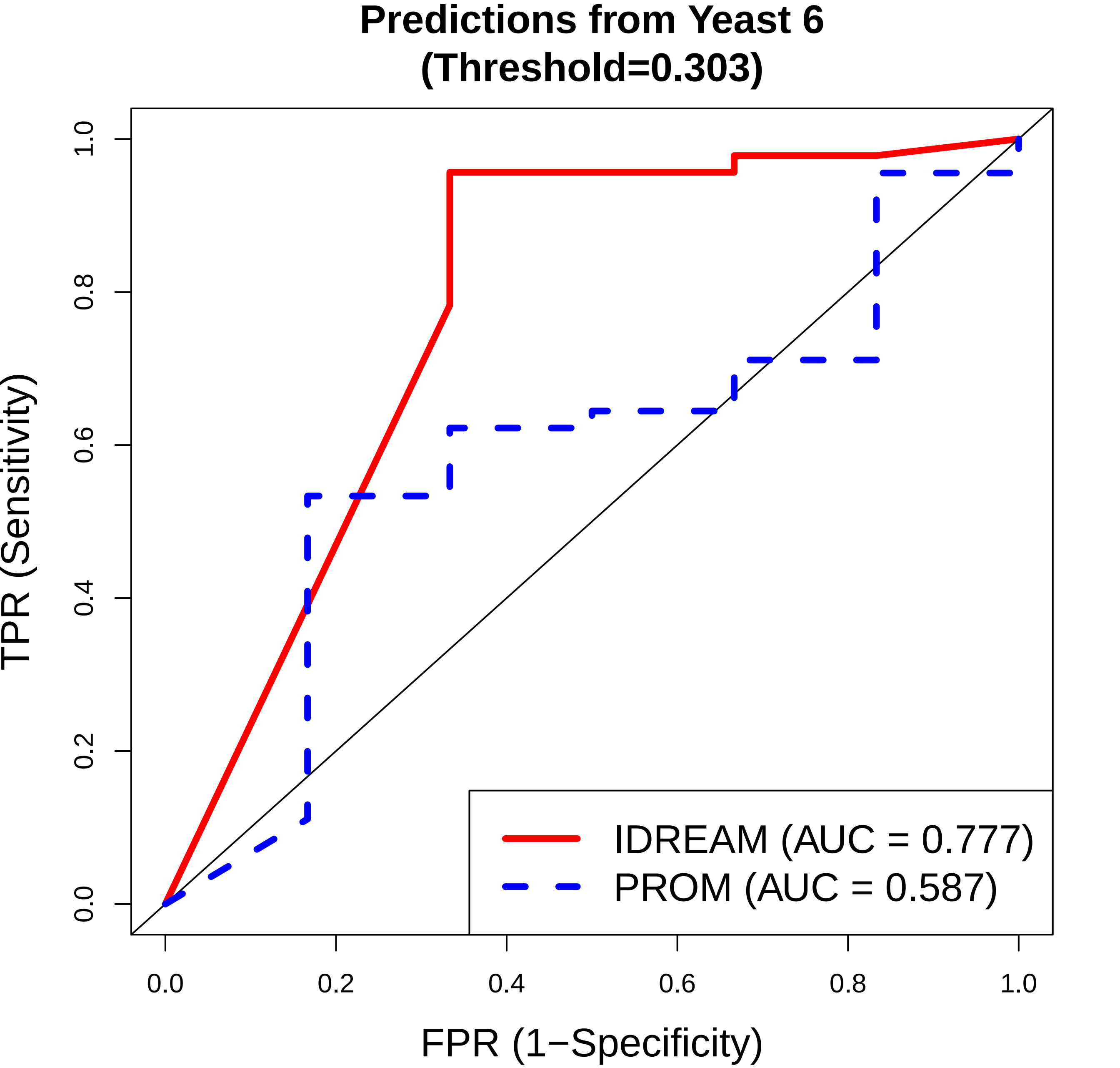

Predictions from Yeast 6
(Threshold=0.303)
TPR (Sensitivity)
FPR (1−Specificity)
0.0
0.2
0.4
0.6
0.8
1.0
IDREAM (AUC = 0.777)
PROM (AUC = 0.587)

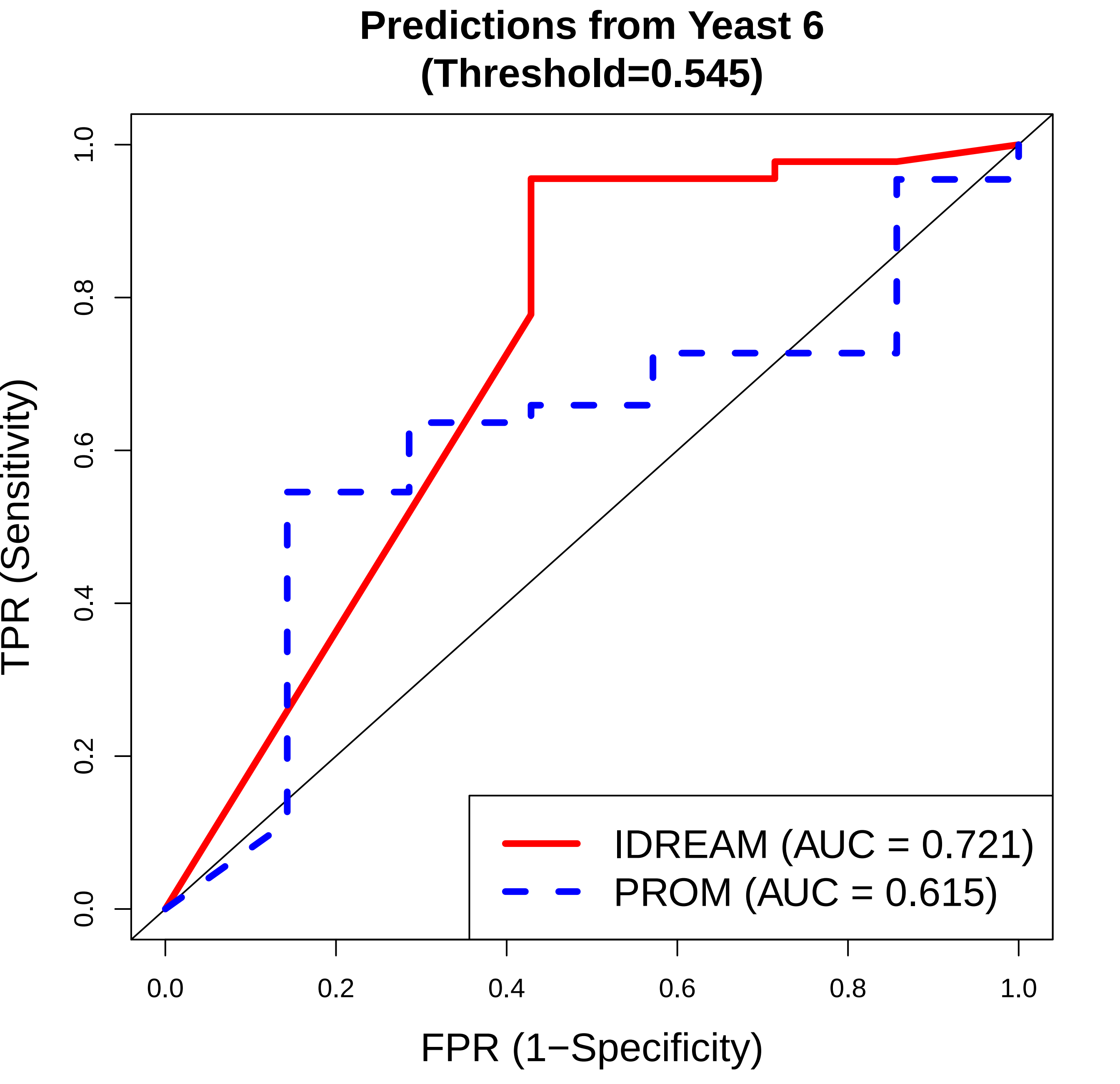
Predictions from Yeast 6
(Threshold=0.545)
TPR (Sensitivity)
FPR (1−Specificity)
0.0
0.2
0.4
0.6
0.8
1.0
IDREAM (AUC = 0.721)
PROM (AUC = 0.615)

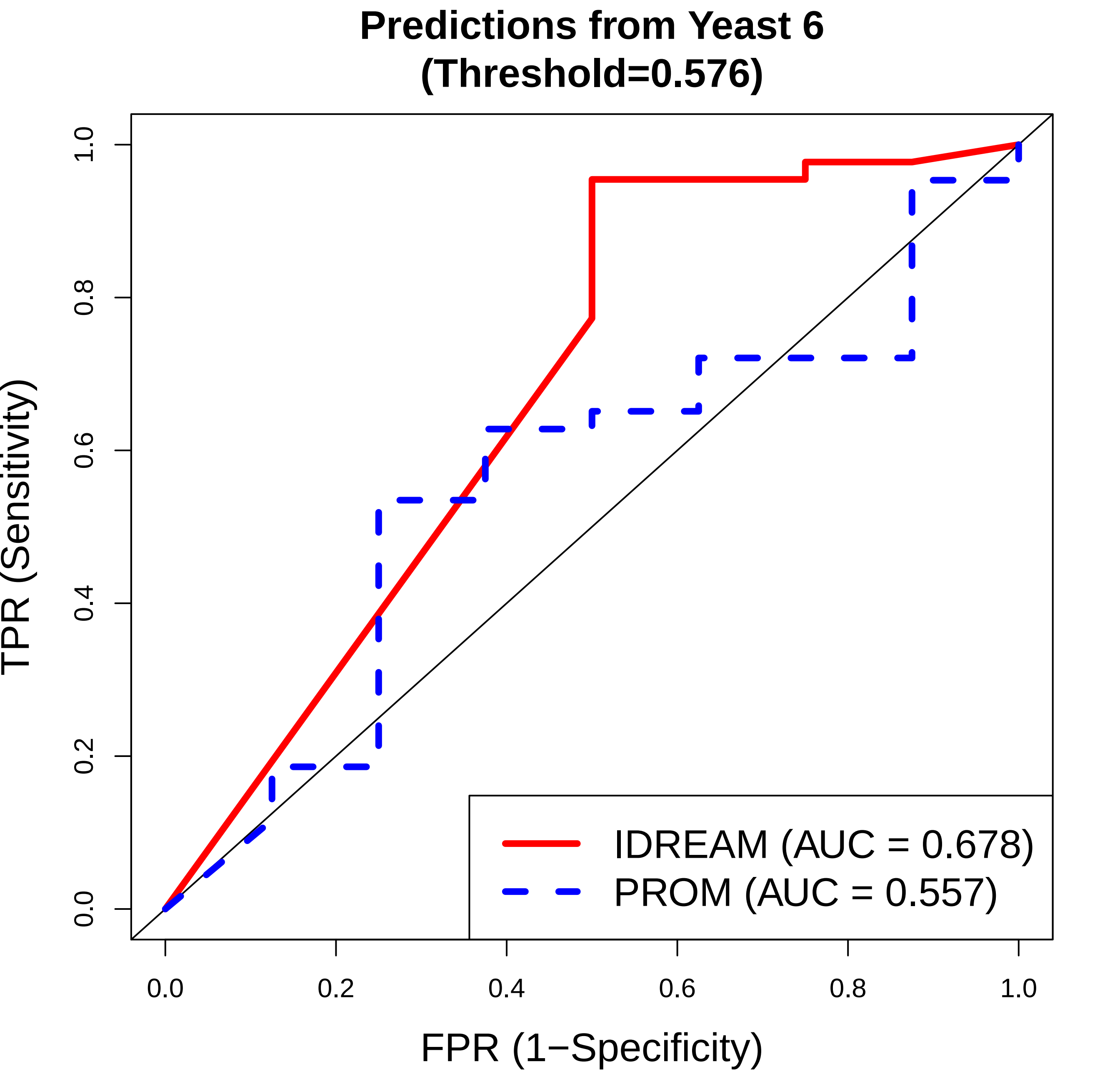

Predictions from Yeast 6
(Threshold=0.576)
TPR (Sensitivity)
FPR (1−Specificity)
0.0
0.2
0.4
0.6
0.8
1.0
IDREAM (AUC = 0.678)
PROM (AUC = 0.557)

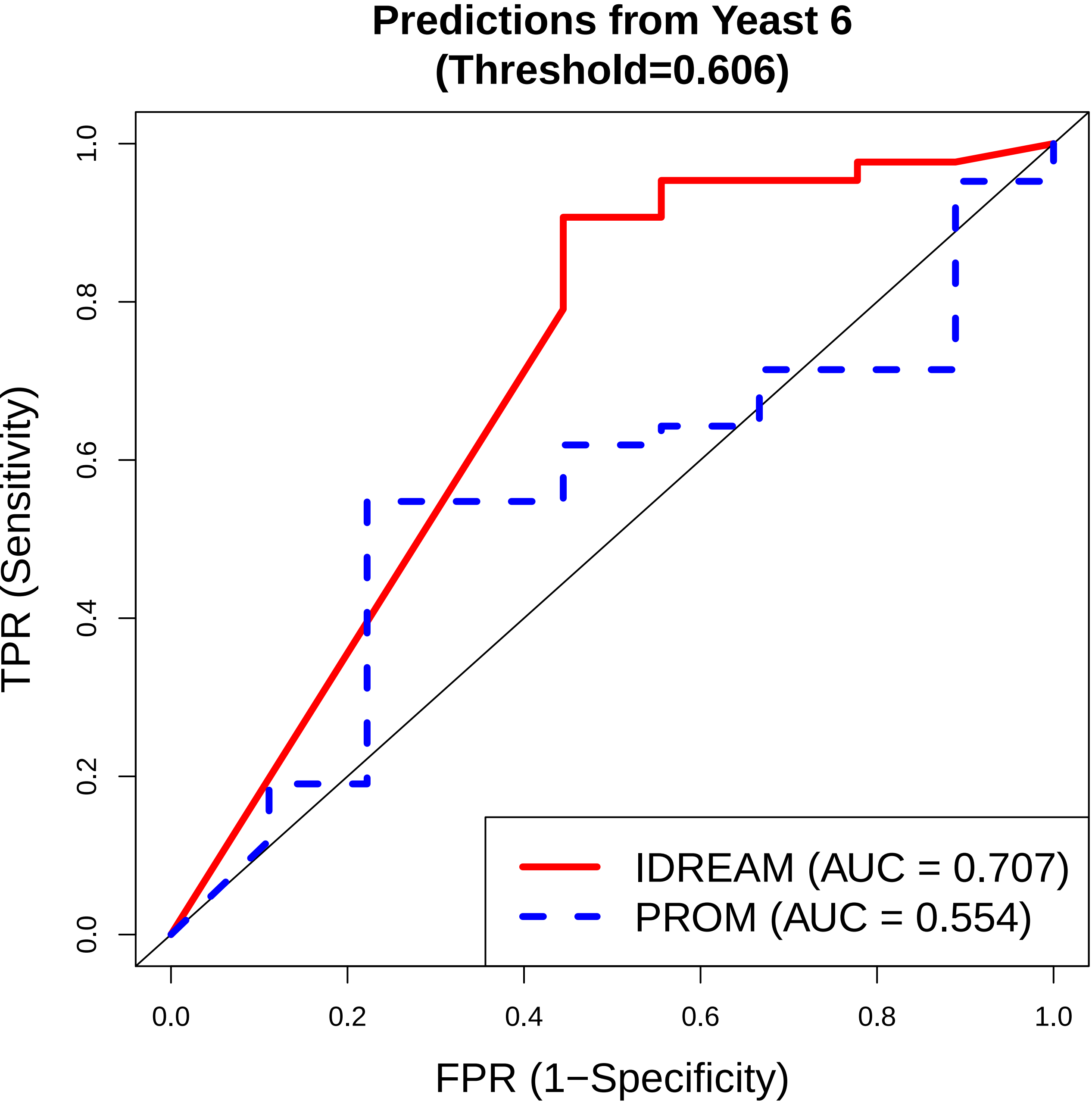
Predictions from Yeast 6
(Threshold=0.606)
TPR (Sensitivity)
FPR (1−Specificity)
0.0
0.2
0.4
0.6
0.8
1.0
IDREAM (AUC = 0.707)
PROM (AUC = 0.554)

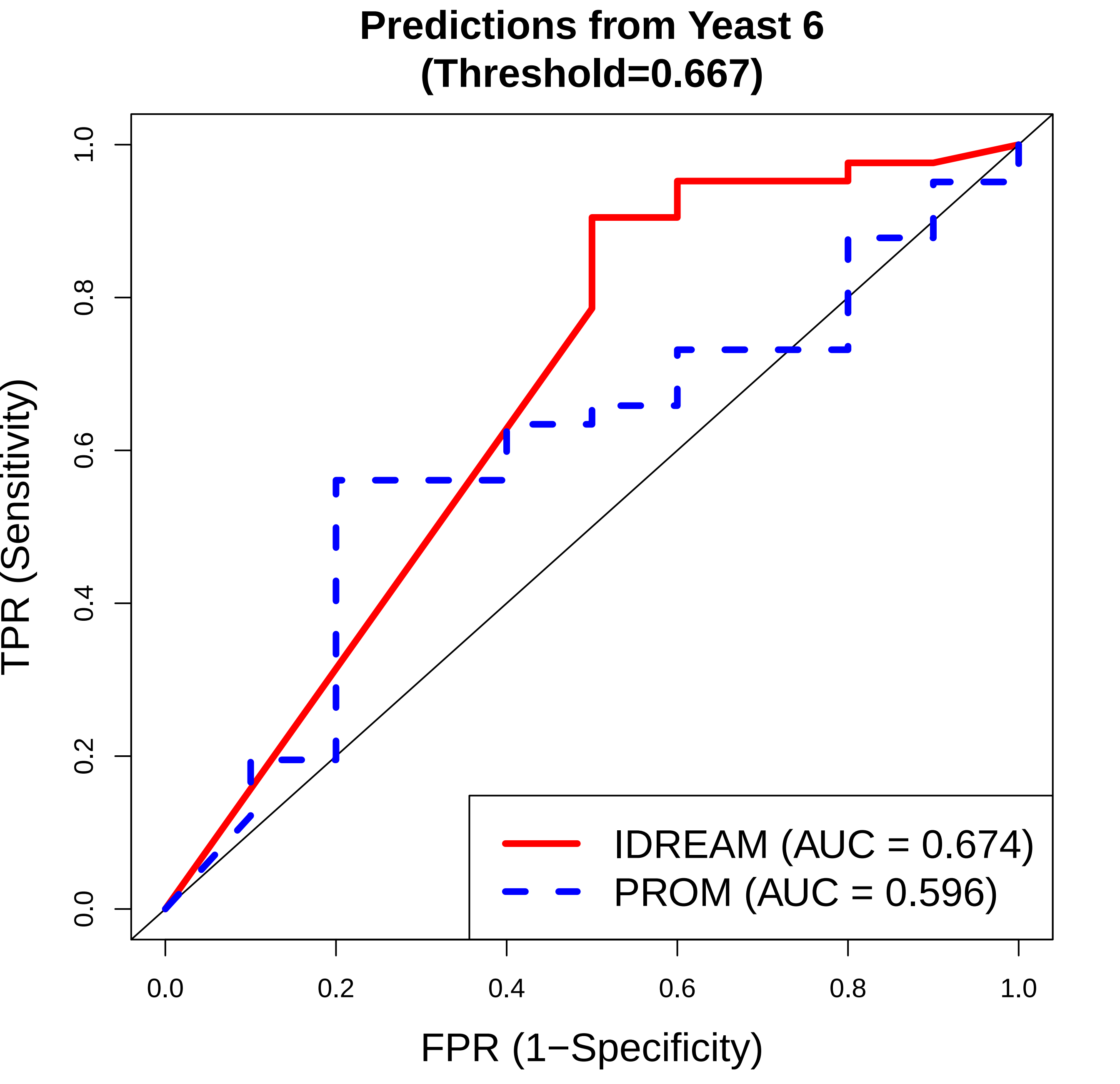

Predictions from Yeast 6
(Threshold=0.667)
TPR (Sensitivity)
FPR (1−Specificity)
0.0
0.2
0.4
0.6
0.8
1.0
IDREAM (AUC = 0.674)
PROM (AUC = 0.596)

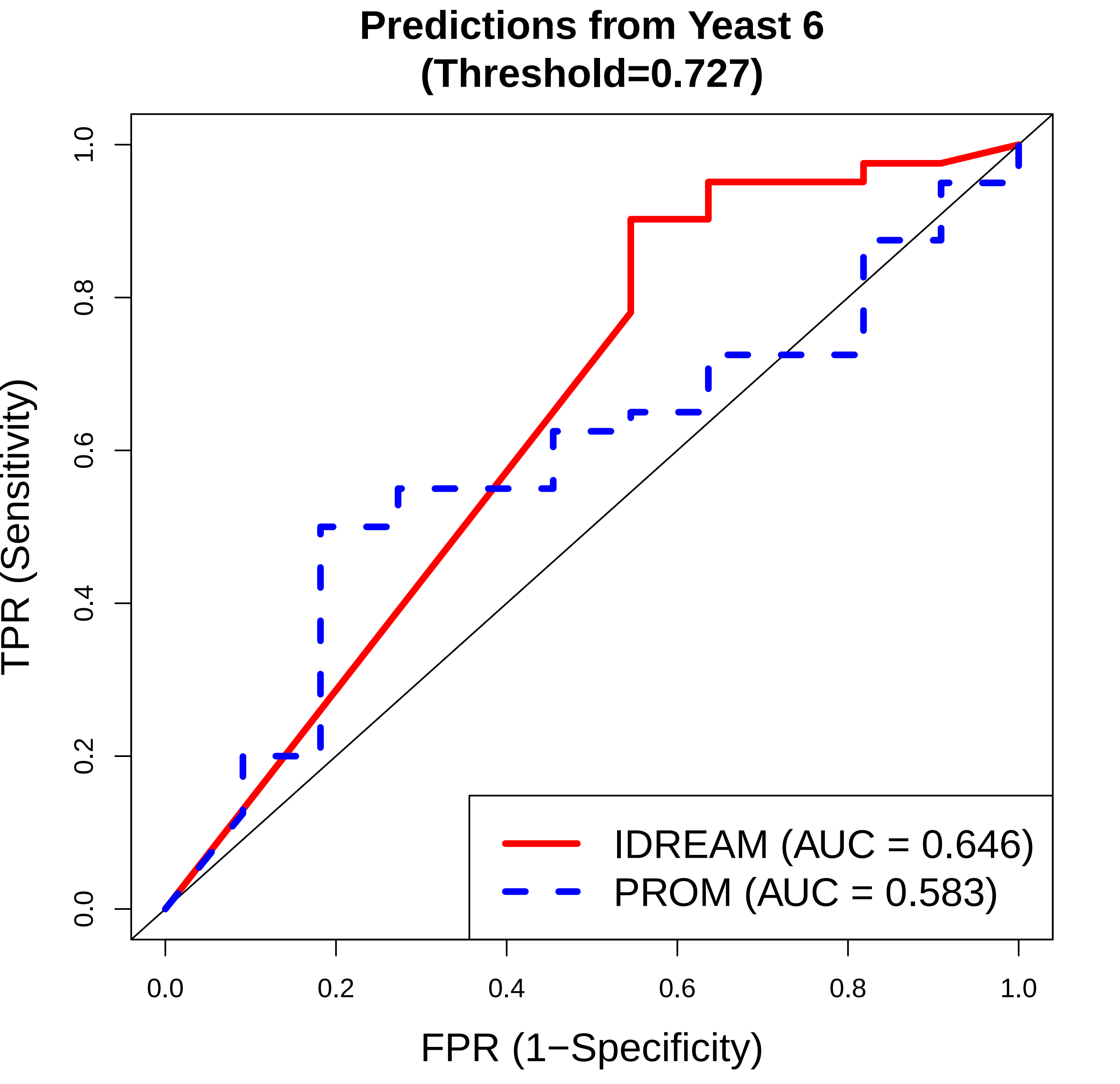

Predictions from Yeast 6
(Threshold=0.727)
TPR (Sensitivity)
FPR (1−Specificity)
0.0
0.2
0.4
0.6
0.8
1.0
IDREAM (AUC = 0.646)
PROM (AUC = 0.583)

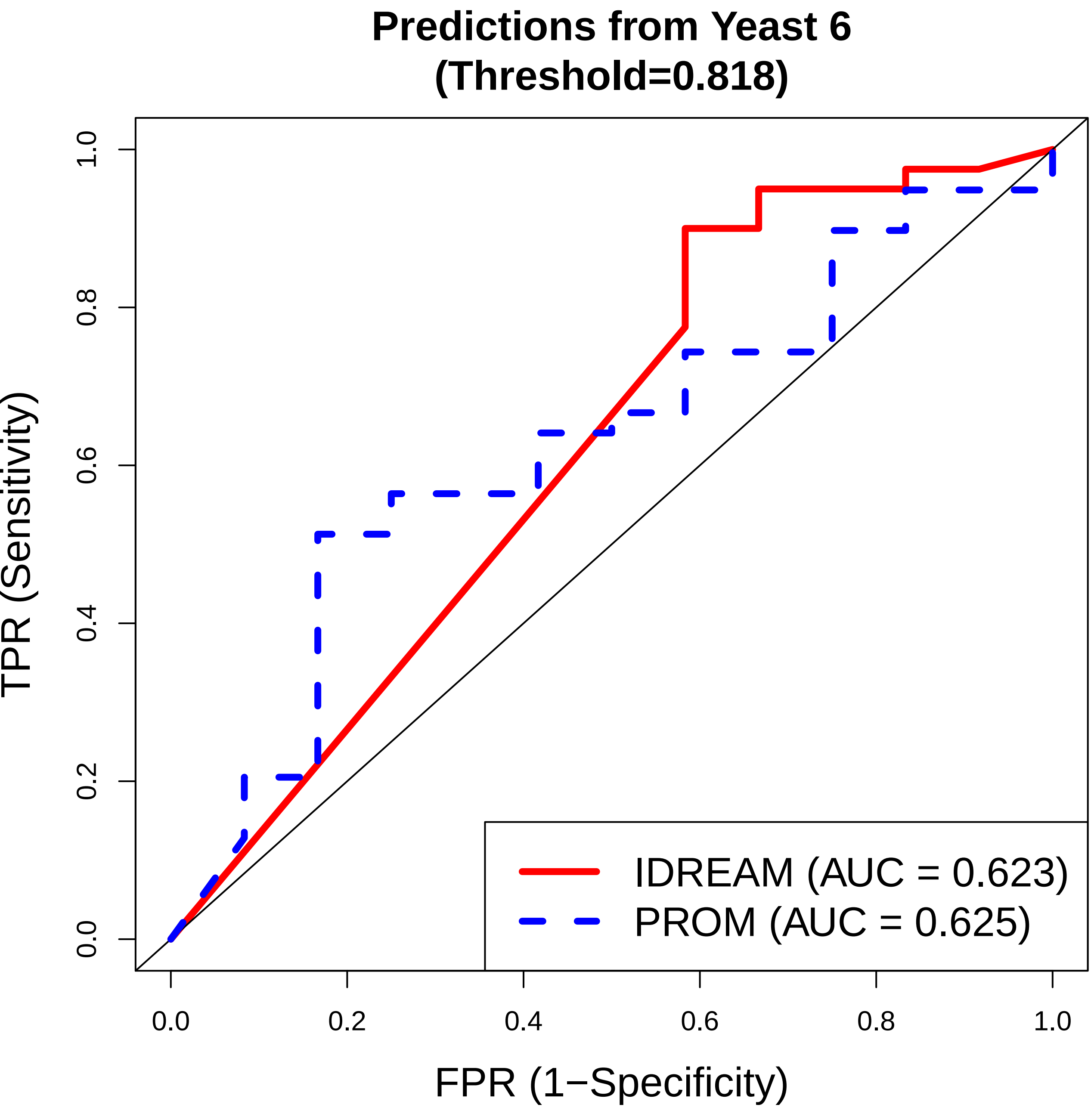

Predictions from Yeast 6
(Threshold=0.818)
TPR (Sensitivity)
0.0
0.2
0.4
0.6
0.8
1.0
FPR (1−Specificity)
IDREAM (AUC = 0.623)
PROM (AUC = 0.625)

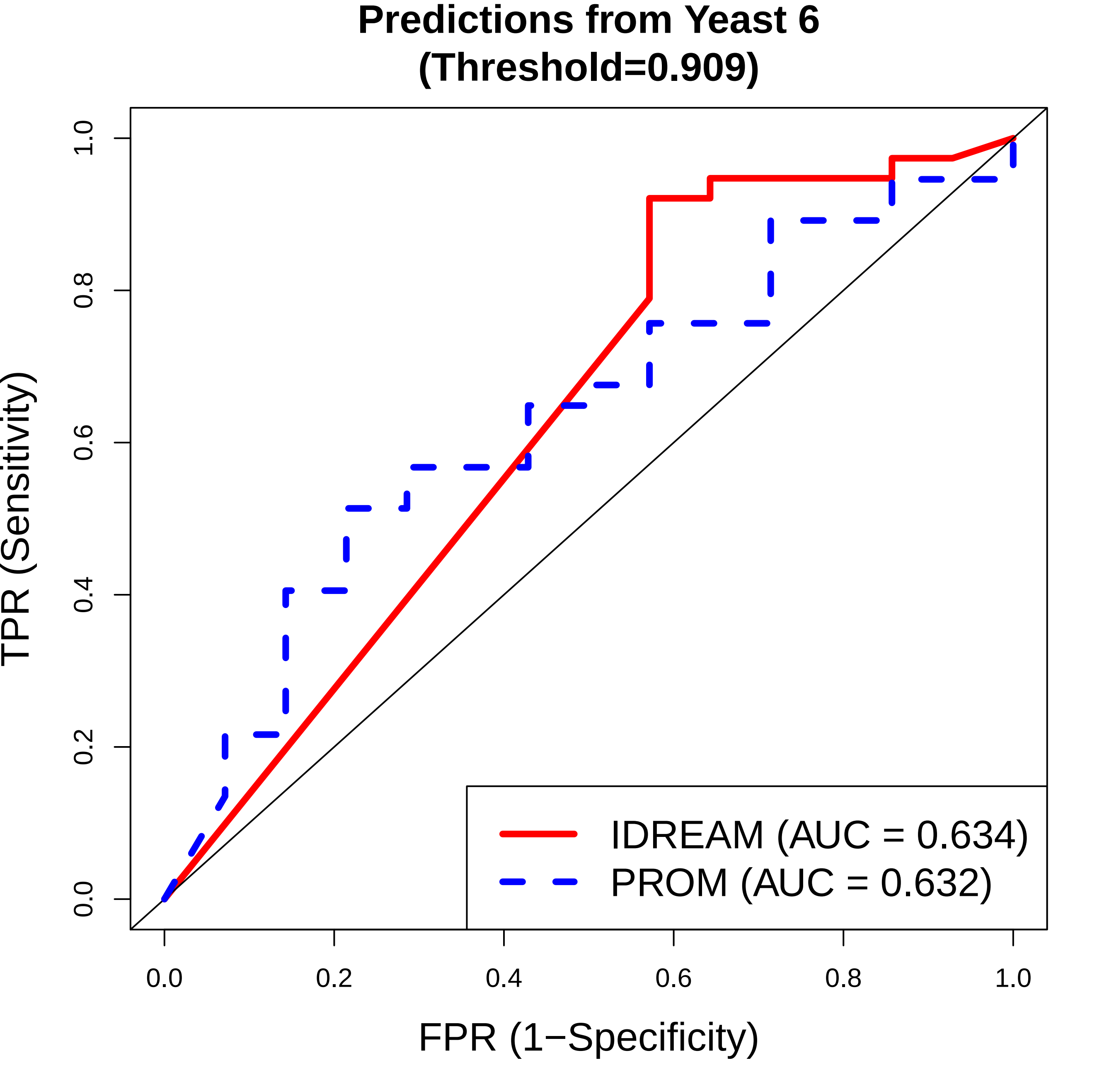
Predictions from Yeast 6
(Threshold=0.909)
TPR (Sensitivity)
FPR (1−Specificity)
0.0
0.2
0.4
0.6
0.8
1.0
0.0
0.2
0.4
0.6
0.8
1.0
IDREAM (AUC = 0.634)
PROM (AUC = 0.632)

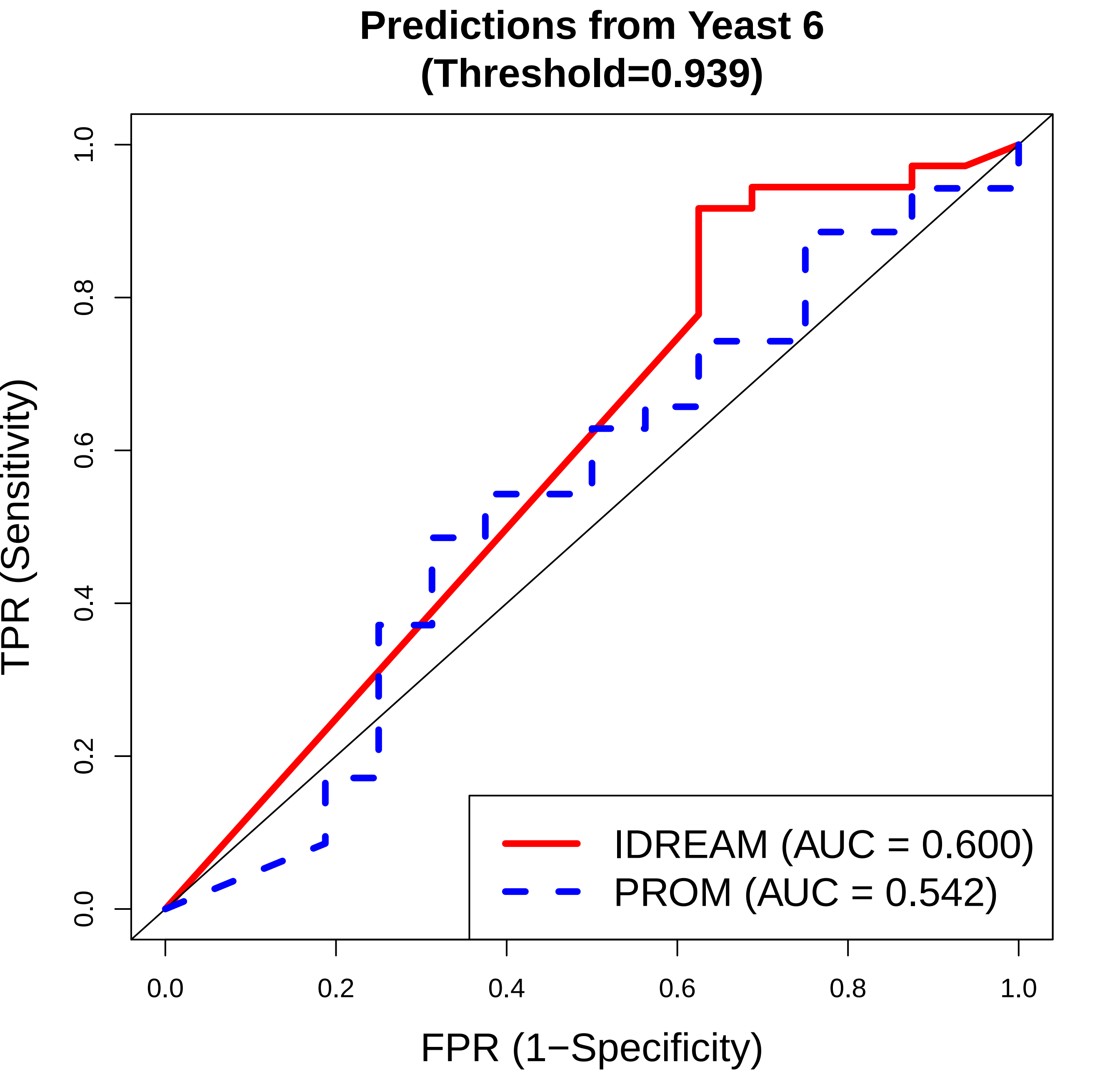
Predictions from Yeast 6
(Threshold=0.939)
TPR (Sensitivity)
FPR (1−Specificity)
0.0
0.2
0.4
0.6
0.8
1.0
IDREAM (AUC = 0.600)
PROM (AUC = 0.542)

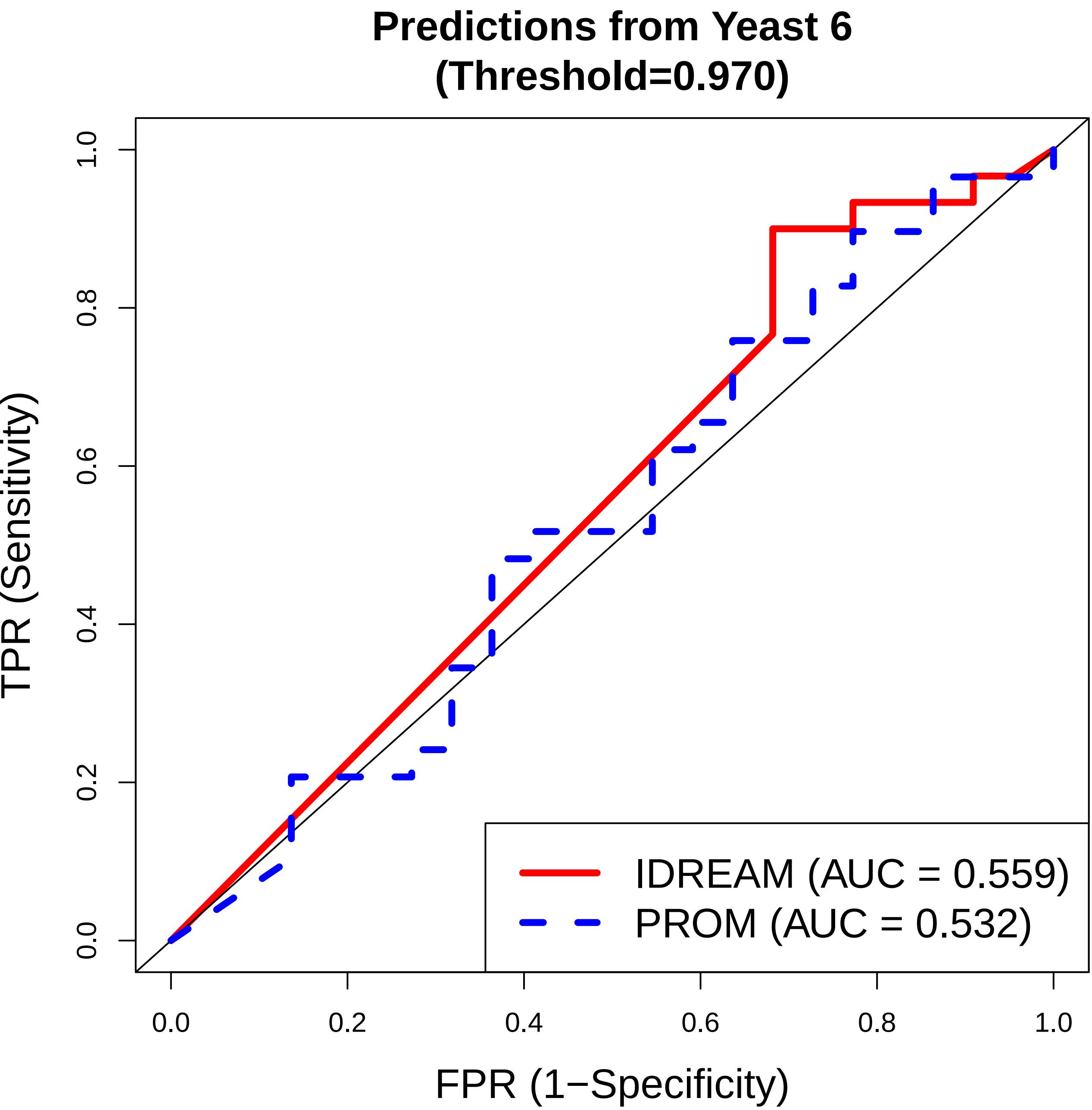
Predictions from Yeast 6
(Threshold=0.970)
TPR (Sensitivity)
FPR (1−Specificity)
0.0
0.2
0.4
0.6
0.8
1.0
IDREAM (AUC = 0.559)
PROM (AUC = 0.532)

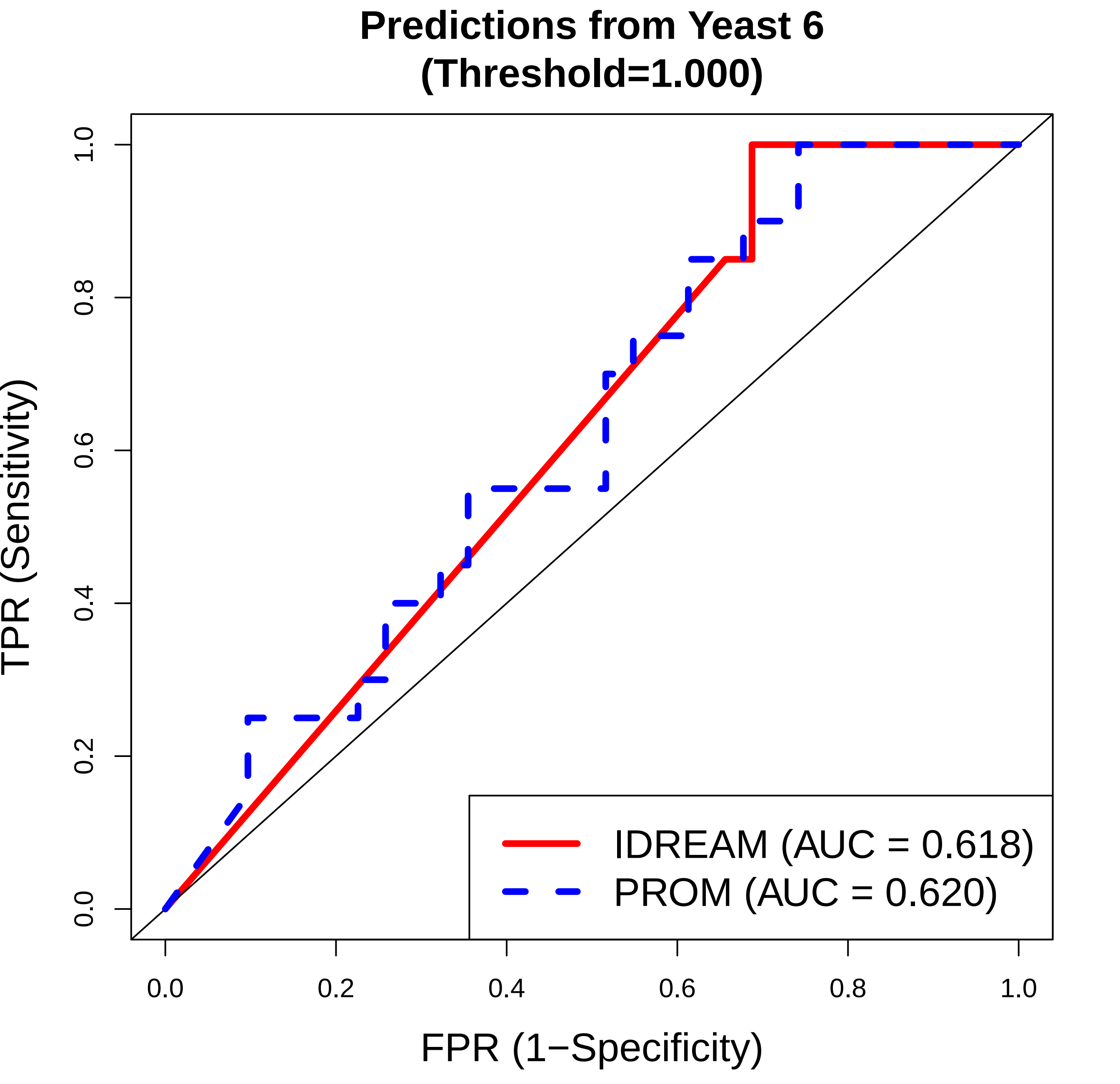

Predictions from Yeast 6
(Threshold=1.000)
TPR (Sensitivity)
FPR (1−Specificity)
0.0
0.2
0.4
0.6
0.8
1.0
0.0
0.2
0.4
0.6
0.8
1.0
IDREAM (AUC = 0.618)
PROM (AUC = 0.620)

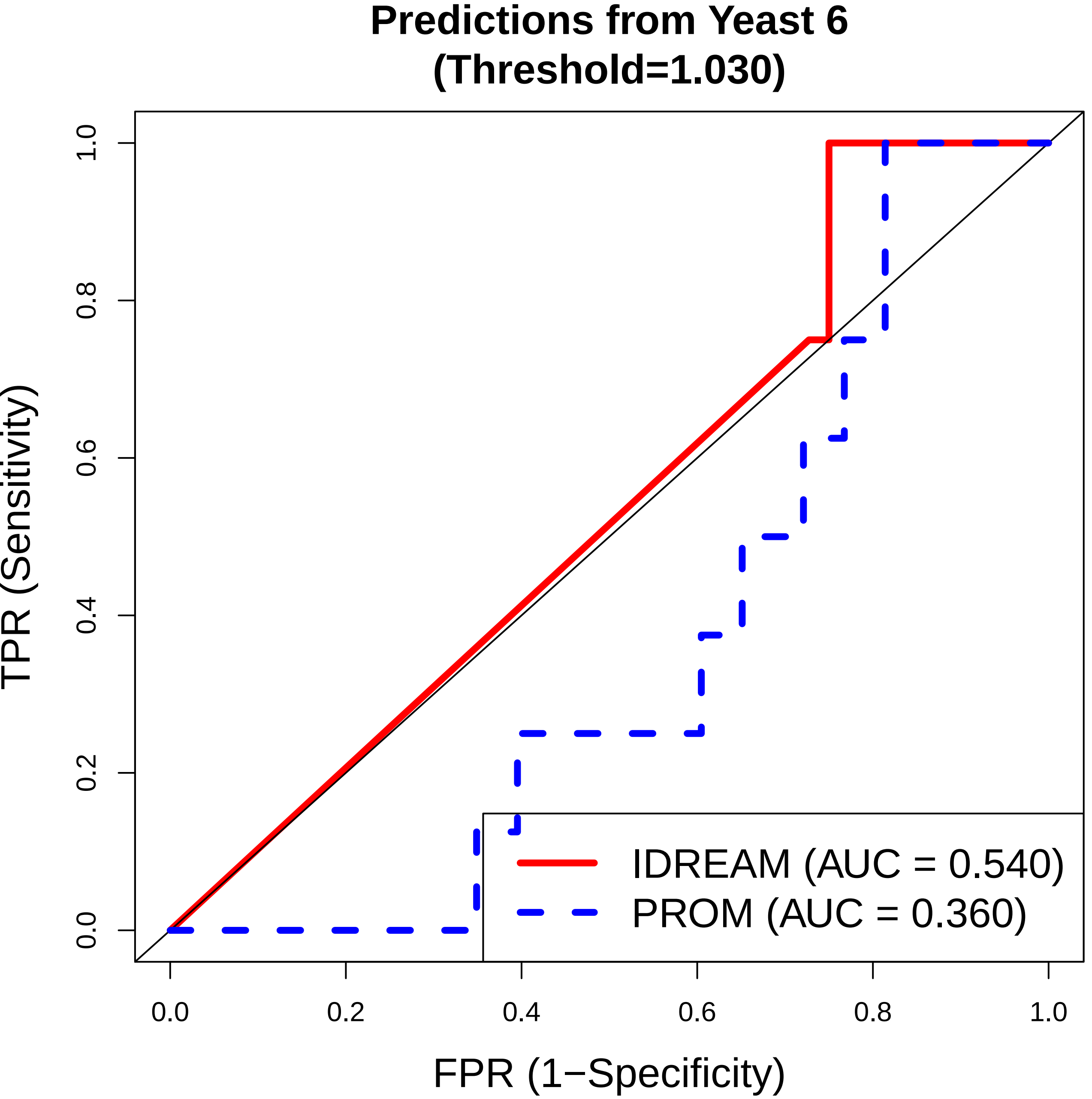
Predictions from Yeast 6
(Threshold=1.030)
TPR (Sensitivity)
FPR (1−Specificity)
0.0
0.2
0.4
0.6
0.8
1.0
IDREAM (AUC = 0.540)
PROM (AUC = 0.360)
