# Supplementary material for: Combining inferred regulatory and reconstructed metabolic networks enhances phenotype prediction in yeast
Source: PLoS Comput Biol. 2017 May 17;13(5):e1005489. doi: 10.1371/journal.pcbi.1005489 (PMC5453602; doi:10.1371/journal.pcbi.1005489)
Supplement: S5 Table — (DOCX) [file pcbi.1005489.s010.docx]

Table S4. Growth predictions with IDREAM by aggregate correlation.

Each row shows the Pearson correlation coefficient between predicted and actual growth. Aggregate refers to the predictions for all three metabolic models taken together. ‘vs. PROM’ shows the difference in aggregate correlations between the IDREAM models and the PROM model. P-values were calculated using a Fisher’s Z transform.

|  | Yeast 6 | Yeast 7 | iMM904 | Aggregate | Aggregate  p-value | *vs.* PROM | *vs.* PROM  P-value |
| --- | --- | --- | --- | --- | --- | --- | --- |
| PROM | 0.1712 | 0.1386 | 0.2261 | 0.1781 | 2.76e-2 | - | - |
| IDREAM-hybrid | 0.4183 | 0.1733 | 0.3202 | 0.2973 | \| 1.64e-4 \| \| --- \| | 0.1192 | 6.97e-2 |
| IDREAM | 0.4325 | 0.2724 | 0.3689 | 0.3546 | \| 5.56e-6 \| \| --- \| | 0.1765 | 4.77e-2 |
