# Supplementary material for: Combining inferred regulatory and reconstructed metabolic networks enhances phenotype prediction in yeast
Source: PLoS Comput Biol. 2017 May 17;13(5):e1005489. doi: 10.1371/journal.pcbi.1005489 (PMC5453602; doi:10.1371/journal.pcbi.1005489)
Supplement: S1 Script — (ZIP) [file pcbi.1005489.s013.zip › IDREAM pipeline description.docx]

IDREAM pipeline description

(Using yeast as an example)

Step 1 -EGRIN: Run boostrapped Inferelator in R to infer the regulatory network

Inferelator.pkg.R

Input: expression matrix (e.g. 6300 genes, 2929 microarrays)

Output: generate regulatory interactions between regulator and targets with the status of activation or inhibition. The interactions with FDR>0.05 are removed.

Step 2 Probabilities: Calculate the probability that an ON/OFF state in a TF will lead to an ON/OFF state in the target genes, according to FDR and activation/inhibition status from EGRIN.

Set litevidence=1 for direct interactions, and litevidence=0 for indirect interactions. For direct interactions, set prob_prior based on the EGRIN FDR: For activation, prob_prior=FDR; for inhibition, prob_prior=1-FDR. For indirect interactions, infer the probability from gene expression data (IDREAM_hybrid) or leave unconstrained (IDREAM).

Step 3 PROM: Run PROM in MATLAB to predict the growth phenotype for each TF knockout.

function [f,v,status1,lostxns] = prom(model,expression,expressionid,regulator,targets,litevidence,prob_prior)

Input: Model is the metabolic model obtained from COBRA toolbox through readCbmodel command; Regulator and targets represent the interaction pairs inferred from EGRIN; litevidence means high confidence interaction, with 1 for direct interaction and 0 for indirect interaction here; prob_prior represents the probability for the direct interaction.

Output: Predicted growth rate (f) and flux response (v) after knocking out the gene for each regulator in the regulatory network.
